# Default Inheritance in Modified Statements: Bias or Inference?

**DOI:** 10.3389/fpsyg.2021.626023

**Published:** 2021-04-30

**Authors:** Corina Strößner

**Affiliations:** Department of Philosophy II, Ruhr-University of Bochum, Bochum, Germany

**Keywords:** modifier effect, default inheritance, prototype theory, compositionality, rational reasoning

## Abstract

It is a fact that human subjects rate sentences about typical properties such as “Ravens are black” as very likely to be true. In comparison, modified sentences such as “Feathered ravens are black” receive lower ratings, especially if the modifier is atypical for the noun, as in “Jungle ravens are black”. This is called the *modifier effect*. However, the likelihood of the unmodified statement influences the perceived likelihood of the modified statement: the higher the rated likelihood of the unmodified sentence, the higher the rated likelihood of the modified one. That means the modifier effect does not fully block *default inheritance* of typical properties from nouns to modified nouns. This paper discusses this inheritance effect. In particular, I ask whether it is the direct result of composing concepts from nouns, that is, a bias toward “black” when processing “raven”. I report a series of experiments in which I find no evidence for a direct inheritance from composition. This supports the view that default inheritance is rather an inference than a bias.

## 1. Introduction

One of the central questions of cognitive science concerns the status of prototypes. During the twentieth century, it became clear that most of our concepts are not definable in terms of necessary and jointly sufficient features. The late Wittgenstein's discussion of “game” is a well-known example (c.f. Wittgenstein, 1953).^1^ Prototype theory (c.f. Rosch and Mervis, 1975; Rosch, 1978) offered the alternative idea that concepts are determined by prototypes. These are highly central exemplars or summary representations of typical properties associated with the concept. While prototype theory has been a very successful research paradigm within psychology, there remain doubts whether concepts should really be understood in terms of prototypes. One prominent voice against the prototype view was Jerry Fodor. In several philosophical works (e.g., Fodor and Lepore, 1996; Fodor, 1998), he argued that concepts should not be identified with prototypes. He accepts that concepts are *associated to* prototypes but denies that they are part of what the concept essentially is. His main critique is that prototypes lack compositionality. The meaning of composed concepts such as “pet fish” is not a straightforward composition of “pet” and “fish”. Different versions of the compositionality criterium have been developed that are more compatible with prototype concepts (Hampton, 1987; Smith et al., 1988; Hampton and Jönsson, 2012; Strößner, 2020). While these versions depart from a very strict reading of compositionality, they still hold that the typical features of a concept such as “raven” influence complex concepts such as “jungle raven” or “feathered raven”. *Prima facie* the typical properties of the concept (e.g., blackness) are inherited by the complex concepts, unless the modifier speaks against inheritance of the typical property (e.g., “albino raven”).

Connolly et al. (2007) deny such inheritance and thus further expand criticism against the apparent lack of compositionality of prototypes. They investigated generic sentences that ascribe typical but non-analytic properties, for example, “Ravens are black” or “Rings are expensive”.^2^ Their subjects rated such sentences with unmodified and modified nouns. Connolly et al. (2007) discovered that humans tend to judge modified statements (e.g., “Feathered ravens are black”) as less likely to be true than unmodified ones, especially if the modifier is atypical (as in “Jungle ravens are black”). This has been called the *modifier effect*. Apparently people do not “default to the stereotype”, as Connolly et al. (2007, p. 5) call it. The work of Connolly et al. (2007) has inspired further experimental research. The upshot of the empirical work is that the modifier effect is extremely robust (Jönsson and Hampton, 2006, 2012; Gagné and Spalding, 2011, 2014; Hampton et al., 2011; Spalding and Gagné, 2015; Gagné et al., 2017; Spalding et al., 2019; Strößner and Schurz, 2020; Strößner et al., 2020). However, it has also been demonstrated that, even though modified statements are perceived as less plausible, the rated likelihood of an unmodified statement correlates with the rated likelihood of the modified one. This indicates that judgments about the modified concept are not independent of the original unmodified concept.^3^

Another debate revolves around the extent to which default inheritance is rationally expected. Connolly et al. (2007) deny that an inference from “Ravens are black” to “Jungle ravens are black” would be rationally justified. Indeed, the inference lacks logical certainty, unless it really means that *all* ravens are black. Statements that ascribe merely typical properties, however, allow for exceptions and the modified noun might refer to an exceptional subcategory. For example, birds can fly but Antarctic birds cannot fly. Nevertheless, the reasoning from categories to subcategories is often intuitively plausible. As a result, the formalization of such inferences gave rise to a whole branch of research on defeasible reasoning. Reiter (1980) started to develop formal logics of default-based logic for artificial intelligence and many other researchers from different disciplines followed (Pearl, 1988, 1990; Kraus et al., 1990; Gabbay et al., 1995; Veltman, 1996; Schurz, 2005). Though the rational justification of default inheritance is still researched, there is a consensus that at least typical subcategories should inherit typical properties. The corresponding inference scheme is called *cautious monotony* and allows inferring “S are typically P” from “C are typically P” and “C are typically S”. Another famous inference pattern is *rational monotony*. It permits to reason from “C are typically P” and “C are not typically non-S” to “S are typically P”. This rule corresponds to default inheritance to subcategories that are not atypical, for example, from a raven to a female raven. Strößner and Schurz (2020) argue that at least the inference to typical subcategories, that is, cautious monotony, is very reliable and entails almost no risk of deriving a false conclusion. Rational monotony is more risky but often still acceptable. Moreover, even the inference to exceptional categories can be quite reliable if the category members have a large overall similarity to each other (Thorn and Schurz, 2018; Strößner, 2020). For example, “Blind ravens are black” might be acceptable, even though blind ravens are atypical, because the blindness is unrelated to color. Very clearly, however, specific background knowledge should always dominate the judgment: No one should accept “Albino ravens are black”. To sum up, default inheritance is often rationally justified. It is a useful reasoning pattern that allows to draw defeasible conclusions about properties of which one has no specific information.

While some forms of default inheritance should and actually do influence our understanding of modified nouns (i.e., subcategories), the details of this process are unclear. Is default inheritance really an inference in human cognition or rather the result of a prototype-induced bias? Is it a by-product of conceptual composition, that is, the result of forming the concept “jungle raven” from “raven”? Or is it detached from composition and only occurring after the meaning of the modifier noun compound has been processed? I present experimental evidence that shows that default inheritance is easily blocked by knowledge effects. This supports the view that default inheritance does not occur as a result of forming complex concepts and that it is rather an inference than a bias.

The paper proceeds with a presentation of empirical findings, starting with a re-analysis of the data from Connolly et al. (2007). I discuss several effects that were discovered in empirical research and how they can be interpreted on the theoretical level. The following part presents a series of experiments that test whether traces of default inheritance are still found when background knowledge intervenes. The largely negative results suggest that the inheritance is not a direct by-product of composition.

## 2. Typicality in Unknown Subcategories

### 2.1. Connolly et al.—A New Analysis

**Figure 1:**
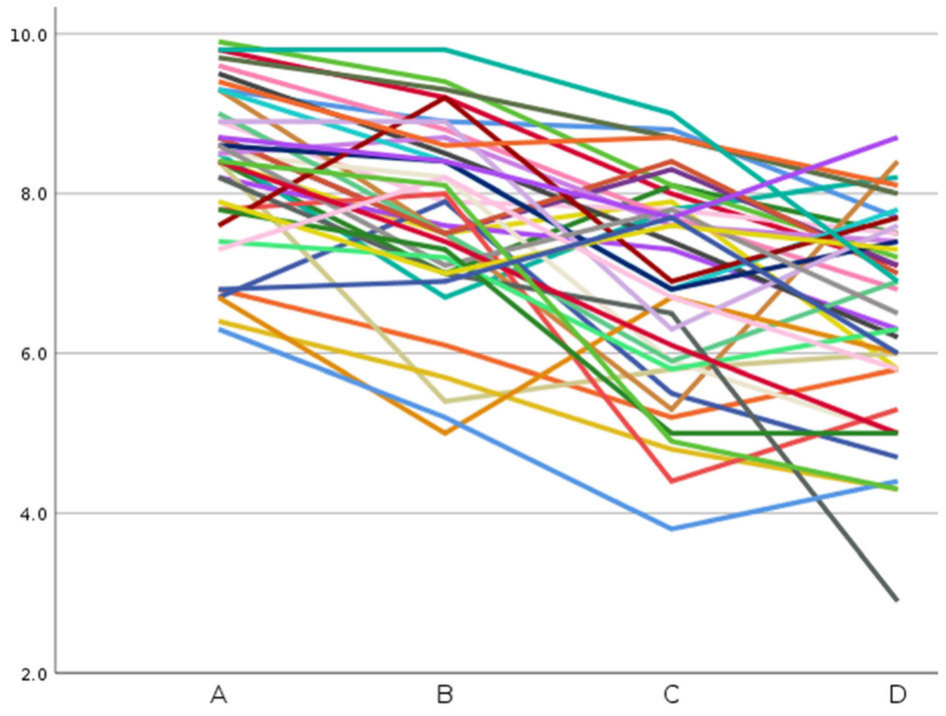
Mean rating of 40 items in Connolly et al. (2007). Each graph corresponds to an item and displays its mean likelihood in the four Conditions A (unmodified), B (typical modifier), C (atypical modifier), and D (additional atypical modifier).

As noted above, research on the modifier effect goes back to an experimental study by Connolly et al. (2007). Their material consisted of 40 items, each with four sentences: an unmodified statement such as “Ravens are black” (Condition A), one with a typical modifier such as “Feathered ravens are black” (Condition B), one with an atypical modifier such as “Jungle ravens are black” (Condition C), and finally a double-modified statement such as “Young jungle ravens are black” (Condition D). Their 40 participants rated one version of each item on a scale from 1 (*very unlikely*) to 10 (*very likely*). Figure 1 provides an overview of the mean ratings of the 40 items in the different conditions.

Connolly et al. (2007) reported the obvious decrease in rated likelihood from condition to condition. They establish its significance by analysis of variance (ANOVA) and pairwise comparisons with *t*-tests. I re-analyzed their data within a mixed-effect model approach, which became the standard method in psycholinguistic research during the last decade because it accounts for the fact that subjects as well as the chosen material are random samples.^4^ This model estimates the mean rating of unmodified conditions as 8.38 (*SE* = 0.2), of typical modifications as 7.72 (*SE* = 0.2), atypical ones as 6.88 (*SE* = 0.2), and of double-modified statements as 6.49 (*SE* = 0.2). All pairwise comparisons are significant (all *p* < 0.001, except atypical and double modification with *p* = 0.003). This conforms with the results reported by Connolly et al. (2007). Moreover, the calculation of model fit indicated a reasonably good model fit (conditional *R*^2^ = 0.332) and a notable but not high effect size of the modifier (marginal *R*^2^ = 0.101).

The decrease effect is quite obvious. However, this does not mean that no inheritance exists. In order to test for the influence of the unmodified statement, I further calculated correlations between the mean rating of the sentences in these different conditions.^5^ Pearson's correlation test revealed that the rating of the unmodified statements was highly correlated with the rating of the typically modified sentences (*r* = 0.71, *p* < 0.001) as well as the atypically modified sentence (*r* = 0.60, *p* < 0.001). The same applies with regard to atypical and double-modified sentences (*r* = 0.62, *p* < 0.001). This speaks against the thesis that the rated likelihood of the unmodified sentences has no influence on the rating of the modified ones and makes it clear that there is not only decrease but also an inheritance effect.

When looking at the individual items, it becomes apparent that the general trend of gradually decreasing probability from A via B to C and D is violated severely by some items (see also Figure 1). A closer view shows that several items might have been affected by common knowledge of the participants. For example, against the general trend, the typically modified statements “Pet hamsters live in cages” and “Jazz Saxophones are made of brass” were judged as more likely (+1.2 and +1.6) compared to their unmodified counterparts. An obvious explanation is that most subjects know that pet rodents are held in cages and that they are acquainted with Jazz saxophones. An example of negative relevance is “flying” in “Flying yellow roosters live on farms” (−3.6). Flying is hardly compatible with being kept on a farm. Another item with potential knowledge effects is “Limousines are long”. The atypical modifier “inexpensive” induced a more drastic loss in rated likelihood than the other items (−4), which points to subject's understanding that smaller cars are less expensive. The further modifier “old” led to an increase in the mean rating (+3.1) indicating that “old” moderates this relation. This search for knowledge effects may seem somewhat speculative, but the crucial point is that it is reasonable to assume that background knowledge influenced the ratings, although Connolly et al. (2007) tried to avoid this in the selection of the material. A thorough analysis of knowledge effects in Strößner et al. (2020) showed that items with potential knowledge effects had significantly greater deviations in the modified conditions.

### 2.2. Aspects of the Modifier Effect

In their discussion, Connolly et al. (2007) primarily focused on the decrease effect: For a concept *C*, prototypical property *T* and the modifier *M*, “*MC* are *T*” is usually rated as less likely than “*C* are *T*”, especially if *MC* is an atypical subcategory. However, their data indicate three aspects:

- Decrease effect: The rated likelihood of “*MC* are *T*” is lower than for “*C* are *T*”.
- Inheritance effect: The rated likelihood of “*MC* are *T*” depends on how likely “*C* are *T*” is.
- Knowledge effect: The rated likelihood of “*MC* are *T*” is strongly influenced by knowledge about *M* or *MC*.

Usually the term “modifier effect” is used to refer to the decrease effect. However, all three effects robustly influence the understanding of modified typicality statements. For example, Jönsson and Hampton (2012) repeated the experiment and reproduced these effects. The modifiers, especially atypical ones, lead to a reduction of the mean rated likelihood (A: 8.31, B: 7.51, C: 6.59, and D: 6.27), but there were also correlations between the judged likelihood of the unmodified and modified statements, which indicates inheritance. Potential influences from knowledge effects were indicated in self-reports by subjects. For example, “Edible catfish have whiskers” was rejected because the whiskers will be removed before eating the fish (c.f. Jönsson and Hampton, 2012, p. 103).

While knowledge may influence the rating of modified nouns, it needs to be stressed that neither the decrease effect nor the inheritance effect is explained by (factual) background knowledge. Gagné and Spalding (2011) replicated the modifier effect for artificial adjectives, that is, pronounceable but meaningless words. This design excludes factual knowledge. In a study by Strößner and Schurz (2020), decrease effects appeared even when subjects mostly denied that the modifier was relevant. However, *if* background knowledge is available, it leads to very strong effects and *tends* to dominate the judgment.

Research has not only established that the modifier effect, especially the decrease effect, is very robust but also that it is more general than initially found by Connolly et al. (2007). It does not only occur for generic statements but also for universal statements such as “All (handmade) sofas have backrests”, even if the universal quantification is emphasized as in “All (handmade) sofas always have a backrest”, “Every single (handmade) sofa has a backrest”, and “100% of (handmade) sofas have a backrest”, as shown by Jönsson and Hampton (2006). Subjects often accept the unmodified universal statements but reject the modified statements, even though the latter are a *logical* consequence of the former. Notably, the effect was weaker in within subjects designs, that is, if the same subjects rated modified and unmodified statements. The effect was further moderated if the sentences were placed beneath each other. Moreover, Hampton et al. (2011) found that the modifier effect is not limited to merely typical properties but equally occurs for analytical properties, for example, in “(Jungle) ravens are birds”. The statement “Ravens are birds” is rated as extremely likely, but the adding of a modifier “jungle” leads to the same amount of decrease as it does in a more contingent statement such as “(Jungle) ravens are black”, where the property is less central (i.e., it is easy to imagine non-black ravens).

As mentioned above, Gagné and Spalding (2011) observed modifier effects even for meaningless words as modifiers. Besides a decrease in rated likelihood, they also noted a longer reaction time (1,406 ms compared to 1,172 ms). Moreover, Gagné and Spalding (2014) replicated these findings for relational sentences instead of modifiers (e.g., “kites that are made of silk” instead of “silk kites”) and even for artificial nouns like “brinn”, when subjects were told that “brinn” refers to a kind of bottle. In Gagné et al. (2017), the hedging words “normal” and “typical” produced a modifier effect. Subjects were told to assume that a generic is true (e.g., “Bottles are cold in annealing ovens”). They were then either asked how many bottles or how many normal bottles or how many typical bottles are cooled in annealing ovens. The mean judgment for the bare noun was 96%, while it was significantly lower for “normal bottles”/“typical bottles” (88%). Spalding and Gagné (2015) also showed that the modifier effect has a reverse sibling. Statements that attribute very *unlikely* properties (e.g., “Whales are small”) are judged as less plausible than their modified counterparts (e.g., “Plary whales are small”). The modifier thus *increases* the judged likelihood of very atypical properties (see also Spalding et al., 2019).

### 2.3. The Role of (Rational) Reasoning

Christina Gagné and Thomas Spalding interpret their findings as evidence against the view that typical properties are directly inherited by subcategories. They deny to view concepts as “containers of properties” such that a modified noun automatically includes the properties as well. According to them, the inheritance is the result of a reasoning process: Participants reason by the meta-knowledge that a subcategory should be somewhat similar and somewhat different. This thesis has the advantage that it explains the inheritance (similarity) as well as the decrease (dissimilarity) as effects of a process that is more or less rationally justified.

However, the *decrease* effect occurs against rational intuitions. For example, rejecting “All handmade sofas have a backrest” but accepting “All sofas have a backrest” as done by subjects in Jönsson and Hampton (2006) is clearly fallacious. Also, it is not clear why central and even categorical properties like “is a bird” are subject to the same amount of decrease. One would expect that people more readily infer categorical properties (like being a bird) than accidental ones (being black).

Much of the apparently irrational effects have been attributed to the particular pragmatic aspects of the task. While logical factors (universal quantifier, essential properties) have little influence on the modification effect, the presentation of the material influences the extent of the decrease effect considerably. For example, placing statements beneath each other leads to a lower decrease effect (Jönsson and Hampton, 2006, 2012). Recently, Strößner and Schurz (2020) showed that the decrease effect was much smaller in a comparative task, where modified and unmodified statements were presented together, as well as in a story-based rating, in which single category members and modifying information were embedded in a story (e.g., about a girl who owns a lamb *Lamby*, a Norwegian lamb *Norwy*, and so on).^6^ In some of their items, the modifier was relevant. Knowledge of positive relevance (e.g., in “Golden rings are expensive”) had a strong effect in the story-based and comparative rating, but not in the normal likelihood rating. The authors conclude that there is still a decrease effect in the background: “In the normal likelihood rating, where not only sentences are evaluated separately, the negative pragmatic effect of the modifier and the positive effect of background knowledge cancel each other out” (Strößner and Schurz, 2020, p. 15). Positive relevance does not prevent a decrease effect but only superposes.

As explanation of the pragmatic effect, Strößner and Schurz (2020) name Gricean implicatures (Grice, 1989). Because people assume that a cooperative speech is as informative and relevant as necessary, the addition of the modifier is automatically perceived as potentially relevant. However, other pragmatic theories such as the relevance theory by Sperber and Wilson (1986) and the more recently developed Rational Speech Act theory (Goodman and Frank, 2016) support a similar prediction that additional information (e.g., a modifier) indicates a meaningful difference. Note that the modified statement is not only longer but takes additional effort in processing: it has a lower fluency. Reber and Unkelbach (2010, p. 568) note a relation between fluency and the relevance theory of Sperber and Wilson (1986), because a lack of fluency also might indicate relevance. A cooperative speaker should make her statements as simple to process as possible.^7^ What appears to be a fallacy in reasoning might thus just be a side effect of otherwise useful cognitive mechanisms.

The pragmatic solution is not totally different from the reasoning approach by Gagné and Spalding. It is even similar to what Gagné and Spalding (2011, p. 189) call the meta-knowledge “that the purpose of using a combined concept is often to refer to a subcategory that is in some way distinct from other members of the head category”. However, Strößner and Schurz (2020) emphasize the *unconscious* nature of the pragmatic component, stating that the *decrease* is not a result of reasoning but of a general relevance bias, which is evolutionarily adaptive but not rationally reflected and of which subjects are not even aware, while Gagné and Spalding leave the status of relevance assumptions open. Their central claim concerns the mechanism behind default inheritance. They criticize a container view of concepts according to which default inheritance is more or less an automatism of conceptual combination (c.f. Gagné et al., 2017, p. 225). Rather, they view inheritance as a result of reasoning.

In what follows, the paper addresses whether inheritance effects should be understood as a result of rational considerations or whether humans are biased toward inheritance just as they are biased toward relevance. To answer the question, I present two experiments that investigate inheritance in the presence of strong knowledge effects and for privative modifier noun combinations (e.g., “stone apple”).

## 3. Experiments

The following experiments aim to address default inheritance in a different way than studies on modification usually do. Most experiments avoid background knowledge. The following experiments do the reverse. I aim to look for inheritance effects when they are not rationally expected. I do this by introducing modifiers with strong negative knowledge effects that should prevent default inheritance. An example is the statement “Dirty pans are used for frying”, where the modifier should prevent inheritance effects.

The experimental idea partly resembles earlier research by Springer and Murphy (1992). They compared modified sentences where a sentence's truth was either determined by the noun alone or was dependent on the modifier. For example, “Peeled apples are sweet” is generally true, while “Peeled apples are white” is true because of the relevant modifier “peeled”. Analogously, the falsity of “Peeled apples are squared” has nothing to do with “peeled”, while “Peeled apples are red” is false because of the modifier “peeled”. It was found that true modified statements are easier and faster to verify if the modifier is relevant, as in “Peeled apples are white” (see also Gagné and Murphy, 1996). Regarding the false sentences, there were no significant differences between generally false statements and those with relevant modifications. The latter finding was cited by Connolly et al. (2007) as evidence against default inheritance. If typicality was inherited, they claim, then sentences such as “Peeled apples are red” should be more difficult to process because “red” would have to be inherited from “apple” and afterwards actively suppressed. However, the experimental design in Springer and Murphy (1992) did not intend to test default inheritance or the modifier effect, which had not been discovered at that time.

As argued above, multiple experiments have shown that the likelihood of “*C* are *T*” has a profound influence on “*MC* are *T*” in the absence of more specific knowledge about *MC*. The aim of the present experiment is to directly assess whether the influence of “*C* are *T*” on the acceptance of “*MC* are *T*” persists if *M* provides strong evidence *against T*. If default inheritance is the result of meta-knowledge or an inference pattern, its influence should be easily blocked if the modifier is sufficiently relevant. In this case, the more specific knowledge should determine the judgment. Thus, it would not be necessary to cognitively rely on usually uncertain default reasoning. If inheritance effects, however, come from a typicality bias or are a mere by-product of composition, their influence should persist.

In order to find these traces of irrational default inheritance, I investigate modified typicality statements with strongly relevant modifiers. However, instead of comparing them to unmodified statements, I compare them to statements with the same modifier but a noun for which no typicality association exists. For example, are there differences between the statement “Peeled apples are red” and “Peeled pears are red” that can be traced back to the fact that “Apples are red” is much more acceptable than “Pears are red”?

The following experimental study starts with a test of unmodified statements with and without typical properties. This is done in the preparatory experiment. An example is the pair of statements “Pans are used for frying” and “Pots are used for frying”. The following two experiments use modifiers with negative knowledge constraints (e.g., “Dirty _____ are used for frying”) and measure how the phrases are evaluated depending on whether the noun is prototypically associated (e.g., “pans”) or unrelated (e.g., “pots”). Measured variables are acceptance (yes/no), reaction time, and a separate plausibility rating. Depending on how deeply people are entrenched to typicality inheritance, the modified sentence “Dirty pans are used for frying” should be still more acceptable than “Dirty pots are used for frying”. An inference-based explanation of modification, on the other hand, predicts that there is no such influence of typicality and that people only rely on the prototype if more specific information is lacking. The effect I am thus mainly investigating is not the decrease effect but the persistence of inheritance effects even if they are not rationally expected.

### 3.1. Preparatory Experiment

My experiment required a set of adequate sentence pairs, consisting of a generic statement that expressed a typical property and a sentence which ascribed the same property to a noun concept for which it is not typical but possible. Apart from the different association to the property, the two nouns should be as similar as possible. I thus constructed 50 sentence pairs (in German) according to the following criteria:^8^

- The noun concepts come from the same superordinate category and have a similar length.
- The typically true statement ascribes a property from the list of associated features by Cree and McRae (2003).
- The other statement ascribes the same property to a noun to which it is usually not associated but still possible.

An example of such a pair is “Rats carry diseases”/“Hamsters carry diseases” (original: “Ratten übertragen Krankheiten”/“Hamster übertragen Krankheiten”). The main purpose of the preparatory experiment was to choose appropriate sentences from the material. Forty subjects were recruited and received payment via the panel Prolific (app.prolific.co). The experiment was programmed and carried out on SoSciSurvey (www.soscisurvey.de).

The material was distributed over two surveys, each with 25 typical and 25 atypical generic statements. Typicality was a between subjects factor. Every participant saw either the true typicality statement or its counterpart. In the first part of the experiment, subjects were presented with the statements and had to decide whether they agree or disagree with the statement as fast and accurately as possible. Reaction time (including reading time) was recorded. In the second part, subjects were allowed to give a more fine-grained judgment on the plausibility of the same statements using a slider (0–100 scale) without any time pressure.

Among the 50 items, I selected 32 pairs that satisfied the following criteria:

- high acceptance of the typical statement, meaning at least 80% of subjects rated “I agree”,
- a considerable difference of acceptability in the atypical and typical statements: acceptance rate of the atypical statement at least 30 points below the rate for the typical statement (e.g., at most 50% if the typical condition received 80% acceptance),
- contingency of the atypical statement, indicated by a plausibility with a mean of at least 10 and a median of at least 5 (on a scale from 0 to 100).

Table 1 displays the least mean squares of the experimental data for the 32 selected items estimated on the basis of a mixed-effect model.^9^ As stipulated, acceptance and plausibility was high for typical generic statements and rather low but not extremely low for atypical generic statements. Moreover, reaction time was longer for the atypical generic statements. The fact that the reaction time of the true generic statements is faster is not unexpected. People are probably highly acquainted with generic statements like “Banana is yellow” and less exposed to statements like “Strawberries are yellow” and this might make them easier to verify and faster to process.^10^

**Table 1:** Least square means of the preparatory experiment.

	**Typical**	**Non-typical**	***R*^2^**
	**Est (SE) [0.95 CI]**	**Est (SE) [0.95 CI]**	**Conditional / Marginal**
Reaction time	1546 (100) [1344, 1746]	1992 (100) [1791, 2193]	0.45/0.06
Acceptance rate	0.96 (0.02) [0.92, 1.01]	0.25 (0.02) [0.21, 0.30]	0.58 / 0.53
Plausibility	87.5 (1.8) [83.9, 91.0]	31.4 (1.8) [27.8, 34.9]	0.64 / 0.58

*Estimated means with standard error in round brackets, 0.95 confidence interval in square brackets and conditional as well as marginal R^2^ in the last column*.

### 3.2. Experiment 1

#### 3.2.1. Methods

*Material:* The material consisted of the 32 sentence pairs from the preparatory experiment with an added modifier that conflicted the ascribed target property. An example is the sentence pair “Heated cellars are cold” and “Heated kitchens are cold” or the aforementioned “Dirty pans are used for frying” and “Dirty pots are used for frying”. The full material is displayed in the Appendix. Additionally, I used 32 true modified sentences. About a half of them were true because of the modifier and the others were true independently of the modifier. Six further fillers were used as warm-up for the reaction time measurement.

*Design:* The 32 sentences with typical noun–property pairs were equally distributed over two questionnaires. Their non-typical counterparts appeared on the other questionnaire, respectively. Moreover, the 38 fillers were added. The experiment consisted of two major parts: a decision task in which participants had to decide as fast and accurately as possible whether they agree or disagree with the presented statements, and a plausibility rating of the same sentences.

*Procedure:* Eighty-two participants were recruited via Prolific and directed to SoSciSurvey, where they were randomly assigned to one of the two questionnaires. In the introductory texts, participants were told that the experiment tests the plausibility of generic sentences without explicitly referring to the notion of typicality. The structure of the experimental procedure was disclosed in the welcome text. That means, subjects were aware that they had to evaluate the same sentences during a decision and a rating task. They were explicitly told that some sentences concern objects of which they have no knowledge and that they should decide intuitively without much thought or research.

During the decision task, participants agreed or disagreed by pressing the buttons 0 or 1.^11^ The next item was presented to them after pressing SPACE. This allowed participants to take self-paced breaks. The decision task was preceded by an instruction and a training run with 10 statements. The experimental block started with six filler questions to avoid warm-up effects. After that, the 32 target sentences and 32 fillers were presented in a random order. Similarly, the plausibility rating task started with a short instruction and a training block. After that, the target sentences and fillers were presented on one page in a random order. At this part of the experiment, subjects were allowed to take as much time as they needed. Other than in the decision task, the survey also allowed for correction of answers.

#### 3.2.2. Results and Discussion

Prior to the analysis, extremely high reaction times (five data points over 15 s) were removed.^12^ An overview of the results can be seen in Table 2. Sentences in which the noun was typically associated to the property were answered faster [β_1_ = −165, *t*_(2505)_ = −4.96, *p* < 0.001]. They also had a slightly higher acceptance rate [β_1_ = 0.04, *t*_(2510)_ = 3.01, *p* = 0.003]. However, the plausibility rating was only insignificantly higher [β_1_ = 1.5, *t*_(2510)_ = 1.80, *p* = 0.07]. All effect sizes were very low, indicating that the typicality did barely influence variation in the data.

**Table 2:** Least square means of Experiment 1.

	**Typical**	**Non-typical**	***R*^2^**
	**Est (SE) [0.95 CI]**	**Est (SE) [0.95 CI]**	**Conditional/Marginal**
Reaction time	2576 (107) [2364, 2789]	2742 (107) [2530, 2955]	0.504/0.005
Acceptance rate	0.19 (0.02) [0.15, 0.24]	0.15 (0.02) [0.11, 0.20]	0.146/0.003
Plausibility	21.2 (2.2) [16.8, 25.6]	19.6 (2.2) [15.3, 24.0]	0.261/0.001

*Estimated means with standard error are in round brackets, 0.95 confidence interval in square brackets, as well as conditional and marginal R^2^ in the last column*.

Let us now look how the reaction time changed in comparison to the preparatory experiment, where unmodified statements were evaluated. Generally, the reaction time was longer, which is expected, because the sentences were now longer and reaction time included reading time. However, the modifiers had a different influence on reaction time for the typical and atypical sentences. The increase on the median reaction time per item was on average 775 ms (*SD* = 362) for sentences without typicality and 1, 007 ms (*SD* = 300) for the sentences with typicality. A paired *t*-test confirmed that the mean difference of 232 ms is significant [*t*_(31)_ = −3.20, *p* = 0.003]. A cognitive mechanism that blocks default inheritance could in principle explain the larger increase in reaction time for sentences with typicality. However, the fact that modified typicality statements were still processed slightly faster than their counterparts speaks against such an interpretation. The more likely explanation is that the typicality statements had an initial processing advantage, which was lost by the added modifier. To check for a potential inheritance effect, I also calculated the correlations between the mean item plausibility rating for typical statements from the preparatory experiment and the ratings of the modified statements in this experiment: no significant correlation was found (*r* = −0.11, *p* = 0.56). The knowledge effects prevented default inheritance.

Another question worth exploring is whether typicality impacted the accuracy of the participants during the fast decision task. In order to address this questions, I detected cases in which the answer during the fast decision task did mismatch the answers in the plausibility rating, where the subjects answered without time pressure and had the option to correct answers. A case was considered to be inaccurate if the participant first accepted the sentence as true but rated its plausibility as lower than 20 or if a sentence was rejected but received a plausibility rate higher than 80. It turned out that the typicality of the noun property pair had no effect on such defined inaccuracy [atypical noun: β_0_ = 0.038; difference for typical noun: β_1_ = +0.004, *t*_(225510)_ = 0.50, *p* = 0.61].^13^

The fact that participants were equally consistent in handling negative relevant knowledge if a typical property noun combination was presented speaks against the thesis that a background inheritance needs to be actively blocked when confronted with relevant knowledge. On the other hand, there was a slightly but significantly higher acceptance rate for statements with typicality. This indicates a minor inheritance effect, even in view of the strongly negative background knowledge of the modifier. The somewhat higher—albeit only almost significant—plausibility values point in a similar direction. Is this the result of a prototype bias or was the negative relevance not perceived as sufficiently strong by the subjects?^14^

The second experiment explores this question by considering privative modifiers, where the modified nouns cannot be interpreted as referring to subcategories (e.g. “stuffed bear”, “paper perl”). In this setting, biases from the noun could persist but a reasoning from categories to subcategories will not occur.

### 3.3. Experiment 2

This experiment investigates whether the effects from experiment 1 occur because the modified noun still refers to a subcategory or whether the noun just triggers an association to the property. If the noun concept's prototype biases participants to associate the property, a slight effect should persist for privative modification, which does not refer to a proper subcategory of the noun category.

#### 3.3.1. Methods

*Material:* The sentence pairs were the same as in experiment 1. However, I now added modifiers that were not only negatively relevant but potentially privative. This means that the modified noun did not refer to a proper subcategory of the noun concepts, for example, “Paper pearls are expensive” and “Paper marbles are expensive”. The full material is again presented in the Appendix.

*Design:* The design resembled that of experiment 1.

*Procedure:* The subjects were recruited and rewarded via Prolific. Overall, 82 persons participated in this part of the study.

#### 3.3.2. Results and Discussion

As in experiment 1, I checked for undue long reaction times and removed one data point over 15 s. An overview of the outcome is given in Table 3, which presents the least square means of the dependent variables.^15^ The noun's association to the property had no significant effect on reaction time [β_1_ = −43, *t*_(2509)_ = −1.38, *p* = 0.169], acceptance [β_1_ = 0.02, *t*_(2510)_ = 1.21, *p* = 0.225], or plausibility [β_1_ = −1.0, *t*_(2510)_ = −1.18, *p* = 0.238]. As before, I checked for inconsistent answers, that is, cases in which a subject accepted a statement but judged its plausibility to be below 20 or rejected the statement but gave a plausibility score over 80. Again, typicality did not influence inconsistency [β_0_ = 0.050; difference for typical nouns: β_1_ = +0.012, *t*_(2541)_ = 1.45, *p* = 0.146].^16^

**Table 3:** Least square means of experiment 2.

	**Typical**	**Non-typical**	***R*^2^**
	**Est (SE) [0.95 CI]**	**Est (SE) [0.95 CI]**	**Conditional/Marginal**
Reaction time	2397 (85) [2227, 2567]	2441 (85) [2271, 2610]	0.384/0.000
Acceptance rate	0.17 (0.02) [0.12, 0.22]	0.16 (0.02) [0.11, 0.21]	0.172/0.000
Plausibility	15.0 (2.1) [10.9, 19.20]	16.0 (2.1) [11.9, 20.18]	0.236/0.000

*Estimated means with standard error are in round brackets, 0.95 confidence interval in square brackets, as well as conditional and marginal R^2^ in the last column*.

The correlation between the mean plausibility rating of the typical statements from the preparatory experiment and this experiment was not significant (*r* = 0.25, *p* = 0.17). Compared to the time measured for the unmodified sentences in the preparatory experiment, the effect of the modifier on the reaction time was different depending on whether the noun and property were associated. For typical nouns, the increase (887 ms, *SD* = 277) was higher than for atypical nouns (534 ms, *SD* = 410). The difference of 353 ms was highly significant [*t*_(31)_ = 4.60, *p* < 0.001]. In view of the other results, it seems unlikely that the additional time is needed to block a default inheritance. Rather, by adding the additional privative modifier, the sentence with a typical noun–property association lost its cognitive advantage and, thus, was processed just as a sentence without any involvement of typicality.

### 3.4. Discussion

**Figure 2:**
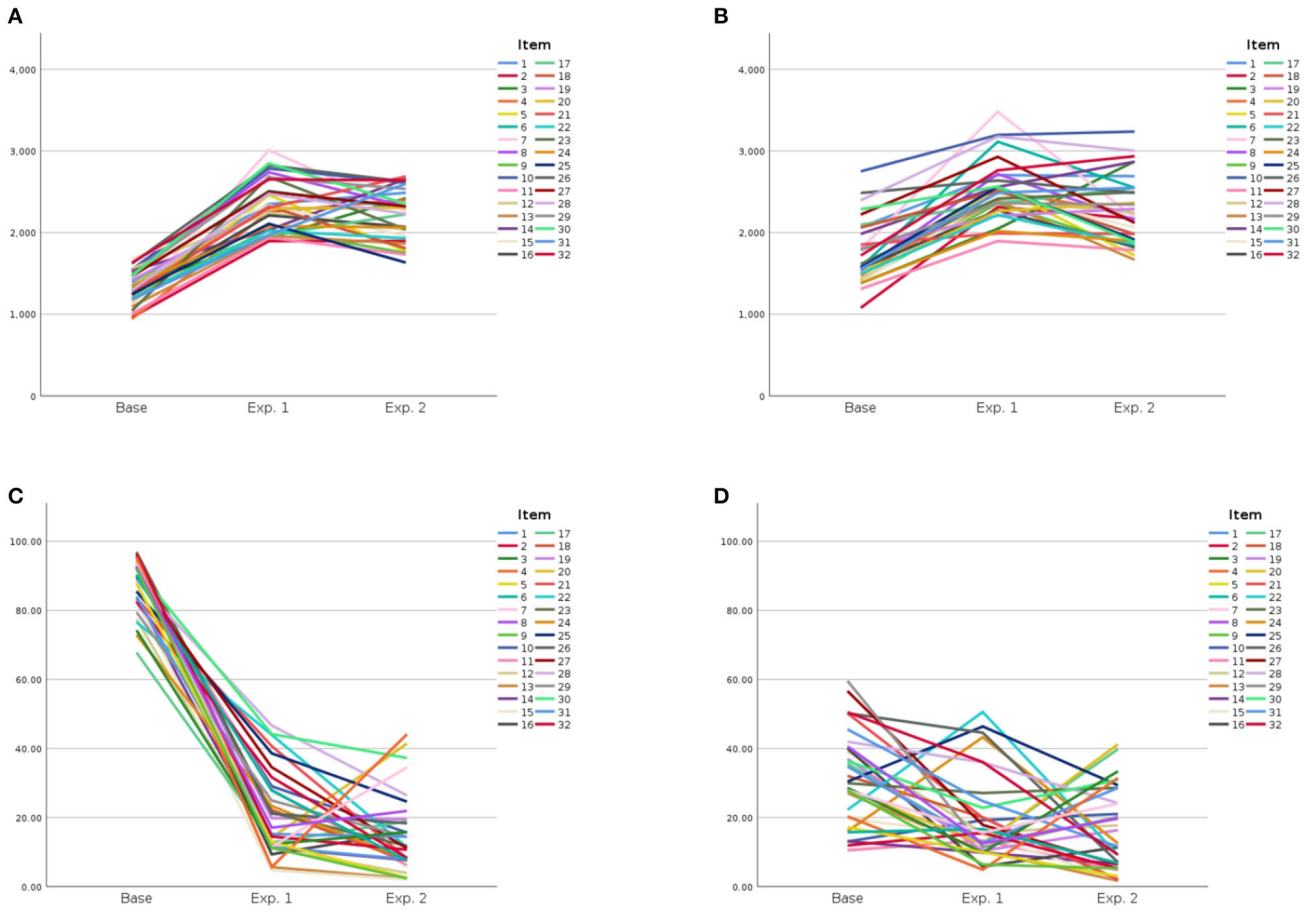
Mean reaction time and mean plausibility of the items in the different experiment, where each line represents the trend of one item. **(A)** Reaction time: typical statements. **(B)** Reaction time: atypical statements. **(C)** Plausibility: typical statements. **(D)** Plausibility: typical statements.

Figure 2 provides a summary representation of the mean item trends over the different experiments. It is quite obvious that the typical statements were processed faster and rated as more plausible in the preparatory experiment, as seen on the left of Figures 2A,C. The adding of relevant (Exp. 1) or even privative (Exp. 2) modifiers lead to a profound increase in the reaction time and decrease in rated plausibility. This is just as one would expect in view of the strong knowledge influences that were introduced by these modifiers.

More interestingly and perhaps surprisingly, the typical noun–property association was *fully* canceled by the knowledge. In comparison to the statements with typicality involvement, the experiments revealed no strong effect of prototypical association between the noun and the target property. Though the acceptance rate was significantly higher for statements with a typical association in experiment 1, the effect was very small. For privative modifications, I found no effect of typicality at all. While it is to be expected that specific knowledge is much more influential than the prototype, the important result is that the prototype did barely influence the judgment at all. If understanding a noun like “raven” presupposes to process typical properties like blackness, it should have been harder to reject statements that mention these properties. However, there is no evidence that subjects were influenced by typicality and that they had to suppress typical properties in order to answer correctly. This becomes especially apparent by the fastness and accuracy of the answers. The results of my experiments thus support one key critique raised by Connolly et al. (2007) and also hold by Gagné and Spalding (2011). There is no evidence that the processing of typical features is necessary in order to understand the complex concepts.

A potential objection to this interpretation is that a lack of evidence of an effect is not equivalent to an evidence of a lacking effect. Indeed, the conclusion I am putting forward here should be viewed with some caution as it essentially rests on negative results. Note, however, that I do not draw the conclusions from the mere lack of statistical significance, which could be easily influenced by the numbers of participants and items. More importantly, the effect sizes in all relevant tests, even those that were significant, are negligibly small. In no way can they explain the considerable default inheritance effect that has been established in the research literature on the modification effect. This makes it very likely that a rational reasoning process—as studied in literature on default logic—lies behind the effect. The gathering of further and more direct evidence for this thesis is an open issue for further research.

## 4. Conclusion

As outlined above, three effects occur if humans are asked to rate the plausibility of a modified sentence: decrease, inheritance, and knowledge effects. Previous research has impressively shown that the decrease effect is extremely stable, even in cases where rational reasoning should block it, that is, for universal statements (Jönsson and Hampton, 2006) or analytic properties (Hampton et al., 2011). Even positively relevant knowledge does not fully block the decrease effect but rather superposes it (Strößner and Schurz, 2020).

The inheritance effect has been less intensively researched than the decrease effect even though it is central for understanding prototype theory to find the source of typicality inheritance. This paper aimed to investigate whether it occurs as a prototype-based bias. The experiments revealed that relevant modifiers tend to block inheritance effects. This result, I conclude, only makes sense if we assume that inheritance occurs as a reasoning process in the absence of knowledge, not as an automatic by-product of composing the meaning. In light of this finding, the reservations Gagné et al. (2017) expressed against a container model of concepts gain support. There is no evidence that we necessarily process concepts as a bundle of such features.

However, I do not reject that concepts are related to prototypes and that they evolve in a way which makes it possible to associate them to prototypes or typical properties (c.f. Jäger, 2007). Indeed, the whole idea of default inheritance, even as an inference, still presupposes concepts that are associated to typical properties (e.g., “cats” or “birds” rather than “non-cats” or “cat and birds”). One general idea of prototype theory is that concepts capture probabilistic covariances in the world (Rosch, 1978; Schurz, 2012) and this is not called into question by my experiments. With the experimental work of this article, I do not reject all ideas of prototype theory in general. The main point is rather that there is no evidence that the processing of a concept alone presupposes to process its prototype or typical features. In view of the many counter-rational findings concerning the decrease effect, this can be interpreted as an optimistic claim: we are easily fooled by our pragmatic biases, but we are not fooled by prototypes.

## Data Availability Statement

The original contributions presented in the study are included in the article/Supplementary Material, further inquiries can be directed to the corresponding author/s.

## Ethics Statement

Ethical review and approval was not required for the study on human participants in accordance with the local legislation and institutional requirements. The patients/participants provided their written informed consent to participate in this study.

## Author Contributions

The author confirms being the sole contributor of this work and has approved it for publication.

## Conflict of Interest

The author declares that the research was conducted in the absence of any commercial or financial relationships that could be construed as a potential conflict of interest.

## Footnotes

^1^Wittgenstein's original argumentation concerned the German “Spiel”, which is a broader term and arguably even harder to define.

^2^Expressing typicality is one of the central functions of generics sentences (Krifka et al., 1995).

^3^Note that all experimental studies on this issue were undertaken with English or German material. The extent to which these findings are replicable for speakers of other languages, especially outside the Indo-European family, is not researched. Care should thus be taken when considering the results as representative for humans or languages in general.

^4^The reanalysis used R and the packages lme4, lmertest, and performance (Bates et al., 2015; Kuznetsova et al., 2017; R Core Team, 2017; Lüdecke et al., 2020). Subject and items were entered as factors with random intercepts. Modification condition was treated as fixed effect. I thank Andrew Connolly for providing the data from the original study.

^5^Such a test was not part of the analysis in Connolly et al. (2007) but has been carried out in later studies, for example by Jönsson and Hampton (2012) and Strößner and Schurz (2020).

^6^Note however that the ratings were generally low for the story-based task.

^7^Generally, the modifier effect seems to be related to fluency effects. However, this issue is under-researched since most studies are focused on what the modifier effect says about the (prototype) theory of concepts.

^8^The experiment was carried out with German native speakers.

^9^Models were again calculated in R (R Core Team, 2017) with the packages lme4, lmerTest, and performance (Bates et al., 2015; Kuznetsova et al., 2017; Lüdecke et al., 2020). Subjects and items were entered with random intercepts. Estimation of degrees of freedom used Satterthwaite's method.

^10^It is a well-established fact that repetition tends to decrease processing time and increases perceived likelihood (c.f. Hasher et al., 1977; Dechêne et al., 2010; Unkelbach and Rom, 2017).

^11^0 for “rather disagree” and 1 for “rather agree”.

^12^Again, I used R with the packages lme4, lmerTest, and performance. Subjects and items were entered with random intercepts. Degrees of freedom were estimated by Satterthwaite's method. The exclusion of extremely long reaction time improved the model fit drastically from conditional *R*^2^ = 0.156 to conditional *R*^2^ = 0.504. Stricter exclusion rules did not further improve model fit.

^13^The mixed effect model was defined as above: item and subject with random intercepts. A more relaxed threshold (accepted, but rated as less than 50; or not accepted, but rated as more than 50 in plausibility) did not affect this general finding [atypical noun: β_0_ = 0.104; difference for typical noun: β_1_ = +0.006, *t*_(2510)_ = 0.52, *p* = 0.60]. Models that merely considered negative deviation (i.e., acceptance but low probability) lead to similar results.

^14^Especially one item in the experiment still received quite high acceptance “Daredevil tortoises are long-living”.

^15^Subjects and items were again entered with random intercepts. Estimation of degrees of freedom is done using Satterthwaite's method. As before, models were calculated in R with the packages lme4, lmerTest, and performance.

^16^The mixed effect model was specified as above: random intercepts for items and subjects. Satterthwaite's method was used for estimation of degrees of freedom.

## Appendix

**Table A1:** Target items of the experiments.

**Typicality statement**	**Paired noun**	**Ex 1 modifier**	**Ex 2 modifier**
Aschenbecher sind schmutzig.	Kafeebecher	Abgewaschen	Essbare
Bananen sind gelb.	Erdbeeren	Verfault	Unsichtbar
Keller sind kalt.	Küchen	Beheizt	Computeranimiert
Raben sind schwarz.	Spatzen	Albino	Marmor
Bären leben im Wald.	Enten	Eingefangen	Ausgestopft
Betten werden zum Schlafen genutzt.	Stühle	Ausgestellt	Zerlegt
Mais wächst auf Feldern.	Pilze	Gewächshaus	Synthetisch
Geschirrspüler stehen in der Küche.	Waschmaschinen	Unverkauft	Zerstört
Delfine leben im Meer.	Schwäne	Eingesperrt	Plastik
Garagen werden zum Parken genutzt.	Lauben	Abgesperrt	Eingerissen
Gorillas sind stark.	Mäuse	Krank	Porzellan
Trauben schmecken süSS.	Rhabarber	Unreif	Eisern
Grashüpfer springen.	Marienkäfer	Beinlos	Gegrillt
Pistolen werden zum Töten genutzt.	Feuerzeuge	Leer	Unecht
Lämmer sind flauschig.	Schweinchen	Nackt	Schokoladen
Erdbeeren sind saftig.	Karotten	Getrocknet	Stein
Spiegel glänzen.	Wandgemälde	Verschmutzt	übermalt
Löwen leben in Afrika.	Füchse	Zoo	Versteinert
Pfannen nutzt man zum Braten.	Töpfe	Dreckig	Verrostet
Pinguine schwimmen.	Tauben	Betäubt	Geschnitzt
Ratten übertragen Krankheiten.	Hamster	Gesund	Zeichentrick
Perlen sind teuer.	Murmeln	Metall	Papier
Zitronen sind sauer.	Mandarinen	Geschmacklos	Gummi
U-Bahnen sind überfüllt.	Taxis	Nacht	Geister
Schwerter sind gefährlich.	Stöcke	Stumpf	Lego
Panzer werden von der Armee genutzt.	Züge	Ausrangiert	Papp
Krawatten sind formelle Kleidung.	Gürtel	Befleckt	Papier
Toiletten haben eine Spülung.	Waschbecken	Camping	Symbolisch
Tomaten isst man in Salat.	Kartoffeln	Ungewaschen	Pulverisiert
Schildkröten sind langlebig.	Salamander	Draufgängerisch	Elektrisch
Traktoren sind laut.	Kräne	Geparkt	Sandkasten
Dreiräder werden von Kindern benutzt.	Einräder	Riesig	Gläsern
Ashtrays are dirty.	Coffee mug	Washed up	Edibles
Bananas are yellow.	Strawberries	Rotten	Invisible
Cellars are cold.	Kitchens	Heated	Computer animated
Ravens are black.	Sparrows	Albino	Marble
Bears live in the forest.	Ducks	Captured	Stuffed
Beds are used for sleeping.	Chairs	Exhibited	Disassembled
Corn grows in fields.	Mushrooms	Greenhouse	Synthetic
Dishwashers are in the kitchen.	Washing machines	Unsold	Destroyed
Dolphins live in the sea.	Swans	Locked up	Plastic
Garages are used for parking.	Arbors	Locked	Torn down
Gorillas are strong.	Mice	Crane	Porcelain
Grapes taste sweet.	Rhubarb	Immature	Iron
Grasshoppers jump.	Ladybird	Legless	Grilled
Guns are used for killing.	Lighters	Empty	Fake
Lambs are fluffy.	Piggy	Nude	Chocolate
Strawberries are juicy.	Carrots	Dried	Stone
Mirrors are shiny.	Wall painting	Dirty	Painted over
Lions live in Africa.	Foxes	Zoo	Petrified
Pans are used for roasting.	pots	Dirty	Rusty
Penguins swim.	Pigeons	Stunned	Carved
Rats carry diseases.	Hamster	Healthy	Cartoon
Pearls are expensive.	Marbles	Metal	Paper
Lemons are sour.	Tangerines	Tasteless	Rubber
Subways are crowded.	Taxis	Night	Ghosts
Swords are dangerous.	Sticks	Stump	Lego
Tanks are used by the army.	Trains	Discarded	Cardboard
Ties are formal wear.	Belt	Stained	Paper
Toilets have flushes.	Washbasin	Camping	Symbolic
Tomatoes are eaten in salads.	Potatoes	Unwashed	Pulverized
Turtles are long-lived.	Salamanders	Daredevil	Electrical
Tractors are loud.	Cranes	Parked	Sandbox
Tricycles are used by children.	Unicycles	Giant	Glass

**Table A2:** Warm up fillers of experiment 1 and 2.

Talking animals can be found in fairy tales.	Glittering cushions are decorative.
Gilded zebras are striped.	Crumpled handkerchiefs are white.
Lion kings have manes.	Inflatable axes are sharp.

**Table A3:** Plausible filler sentences of experiment 1 and 2.

Brown ants live in the ground.	Fresh salad is green.
Silver apples are round.	Paper boats are light.
Perforated umbrellas have a handle.	Fake cops wear uniforms.
Small blueberries are fruits.	Artificial flowers are durable.
Filterless cigarettes are unhealthy.	Fake certificates are rectangular.
Beautiful crows have feathers.	Slaughtered calves are eaten.
New pens need ink.	Model trains are used by children.
Colorful tents are waterproof.	Water pistols are toys.
Clean benches are used for resting.	Melted rings are hot.
Unfurnished apartments have windows.	Wooden horses have four legs.
Prison beds are uncomfortable.	Waving cats are colorful.
Successful actresses are rich.	Miniature pyramids can be built by oneself.
Angry chimpanzees are loud.	Vegan sausages are edible.
Electric bikes are heavy.	Canned fish is edible.
Public pianos have many users.	Former US presidents are famous.
Carving knives are used in the forest.	Candied nuts are sweet.
